# *GCSscore*: an R package for differential gene expression analysis in Affymetrix/Thermo-Fisher whole transcriptome microarrays

**DOI:** 10.1186/s12864-021-07370-2

**Published:** 2021-02-01

**Authors:** Guy M. Harris, Shahroze Abbas, Michael F. Miles

**Affiliations:** 1 VCU Pharmacology and Toxicology, Richmond, Virginia 23298 USA; 2 VCU Center for the Study of Biological Complexity, Richmond, Virginia 23298 USA; 3 Department of Pharmacology & Toxicology and Neurology, Virginia Commonwealth University, Richmond, VA 23298 USA

**Keywords:** Oligonucleotide microarray, Transcriptomics, Differential expression, Software

## Abstract

**Background:**

Despite the increasing use of RNAseq for transcriptome analysis, microarrays remain a widely-used methodology for genomic studies. The latest generation of Affymetrix/Thermo-Fisher microarrays, the ClariomD/XTA and ClariomS array, provide a sensitive and facile method for complex transcriptome expression analysis. However, existing methods of analysis for these high-density arrays do not leverage the statistical power contained in having multiple oligonucleotides representing each gene/exon, but rather summarize probes into a single expression value. We previously developed a methodology, the Sscore algorithm, for probe-level identification of differentially expressed genes (DEGs) between treatment and control samples with oligonucleotide microarrays. The Sscore algorithm was validated for sensitive detection of DEGs by comparison with existing methods. However, the prior version of the Sscore algorithm and a R-based implementation software, *sscore*, do not function with the latest generations of Affymetrix/Fisher microarrays due to changes in microarray design that eliminated probes previously used for estimation of non-specific binding.

**Results:**

Here we describe the GCSscore algorithm, which utilizes the GC-content of a given oligonucleotide probe to estimate non-specific binding using antigenomic background probes found on new generations of arrays. We implemented this algorithm in an improved *GCSscore* R package for analysis of modern oligonucleotide microarrays. G*CSscore* has multiple methods for grouping individual probes on the ClariomD/XTA chips, providing the user with differential expression analysis at the gene-level and the exon-level. By utilizing the direct probe-level intensities, the GCSscore algorithm was able to detect DEGs under stringent statistical criteria for all Clariom-based arrays. We demonstrate that for older 3′-IVT arrays, *GCSscore* produced very similar differential gene expression analysis results compared to the original Sscore method. However, *GCSscore* functioned well for both the ClariomS and ClariomD/XTA newer microarrays and outperformed existing analysis approaches insofar as the number of DEGs and cognate biological functions identified. This was particularly striking for analysis of the highly complex ClariomD/XTA based arrays.

**Conclusions:**

The *GCSscore* package represents a powerful new application for analysis of the newest generation of oligonucleotide microarrays such as the ClariomS and ClariomD/XTA arrays produced by Affymetrix/Fisher.

**Supplementary Information:**

The online version contains supplementary material available at 10.1186/s12864-021-07370-2.

## Background

Despite the advent of RNAseq for transcriptomics analysis, microarrays continue to be widely used with an average of over 7000 PubMed listings per year in 2015–2019 for this technology. A major commercial platform for microarray analysis, produced originally by Affymetrix and now by Thermo-Fisher, utilizes collections of oligonucleotides distributed across cognate genes to probe RNA expression by hybridization. Popular analysis methods for oligonucleotide arrays, such as the Robust Multiarray Analysis (RMA) method, produce expression values for given genes/transcripts/exons by summarizing hybridization intensities across all corresponding oligonucleotides [1]. Since expression “differences” rather than absolute expression levels are generally the goal in microarray studies, our laboratory previously developed the Sscore algorithm for analysis of Affymetrix oligonucleotide microarrays for detecting significant expression changes between paired samples [2]. This entailed comparing individual oligonucleotide probes within each probeset between two samples, after applying a heteroscedastic error correction model. The Sscore method provided an easily interpretable standard normal distribution of expression differences between two samples for a given probeset, akin to a z-score transformation. Prior work demonstrated the advantage of the Sscore method over probe summarization techniques such as RMA for Affymetrix microarray analysis, particularly for experiments having smaller numbers of replicates [2, 3]. This advantage prompted development of a Bioconductor R package, *sscore*, for application of the original Sscore method on Affymetrix microarrays [4]. This algorithm has been utilized in publications across multiple laboratories for studies based on 3′ IVT array technology [5–12].

The original Sscore relies on the difference between perfect match (PM) and cognate mismatch (MM) probes to correct for non-specific hybridization while calculating a measure of expression difference between two samples. MM probes were designed to capture array background noise (rawQ) and non-specific binding (NSB) of off-target transcripts. However, MM probes were subsequently shown to be an inconsistent measure of non-specific binding [13]. While the RMA method excluded MM probe data from the expression calculation and has come to be widely used, use of RMA summarization/normalization followed by appropriate statistical testing differential gene expression does not utilize probe-level information as with the S-score values.

Since NSB signal is strongly correlated with GC-content of the probe sequence, newer Affymetrix array technology eliminated the MM probes and instead utilized 16,943 antigenomic probes of varying GC-content on 25-mer oligonucleotide targets ranging from 3 (12% GC) to 25 (100% GC) to estimate non-specific binding [14]. This new technology, referred to as Whole Transcriptome (WT) arrays, allows probes to be grouped either in Transcript Cluster IDs (TCids) for a gene-level analysis or into Probe Selection Region IDs (PSRids) for an exon-level analysis. Subsequent arrays designs provide more detailed measures of exon expression and transcript splicing variants, via exon-exon Junction IDS (JUCids), that are on par with RNA-seq [15]. These Transcriptome Assay chip types were released for human (HTA1.0/2.0), rat (RTA1.0), and mouse (MTA1.0). This fully featured design was further developed into the ClariomD chip type, available for human and mouse. An additional gene-level only chip type, the ClariomS, was created using only the ten best performing probes from each TCid present on the ClariomD/Transcriptome Assay (HTA/RTA/MTA) designs, which will be referred to as ClariomD/XTA arrays. Unfortunately, no generations of the WT-style arrays are compatible with the original Sscore algorithm and existing *sscore* R package for probe-level analysis due to lack of MM probes on the new array designs. We have therefore developed an R package, *GCSscore*, with a new algorithm that enables the Sscore probe-level analysis method for the newest generation of Affymetrix/Thermo-Fisher Clariom-style microarrays. We have also utilized updated data handling methods that improve the speed of the analysis considerably. We show here that *GCSscore* performed similarly to the originally published *sscore* R package for older type microarrays (3′ IVT) and added functionality for analysis of the newest Affymetrix/Fisher microarray types. Furthermore, our results suggest that use of the *GCSscore* package provides substantial benefit compared to existing methods in detection of differential gene expression (DEG) on these newer generation microarrays.

## Implementation

### Algorithm

The most fundamental change from the original algorithm is the introduction of background correction based on the median signal of antigenomic probes having the same GC-content as the given PM probe, rather than relying on the cognate MM probe hybridization signal. The GCSscore algorithm is based upon the operations in eqs. (1) through (3). For a given probe grouping method (as defined below), ***k***, which is made up of ***N*** probes, the GCS-score, denoted as ***GCSs***_***k***_, is as follows:

1 $$ {GCSs}_k=\sum \limits_{i=1}^N\frac{l_{iB}-{l}_{iA}}{\varepsilon_I\sqrt{N}} $$

2 $$ {\varepsilon}_i=\sqrt{\gamma^2\left({l}_{iB}^2-{l}_{iA}^2\right)+{SDT}_A^2+{SDT}_B^2} $$

3 $$ SDT=4\ast raw\mathrm{Q}\ast SF $$

In the algorithm equations, *l*_*iA*_ and *l*_*iB*_, represent the background corrected intensities of the i-th probe pair from array A and B, respectively. As defined in the Affymetrix MicroArray Suite (MAS) documentation, the significant difference threshold (SDT) is determined by the noise floor of each array and the chosen scaling factor. The noise floor (rawQ) is calculated from the standard deviation of the bottom 2% of the probe intensities across the array. The scaling factor (SF) for each array is a multiplier that scales the median intensities to a target value (default is 500). The gamma factor is set to 0.1 to prevent calculated GCS-score values from being affected by gene expression levels [2].

The *GCSscore* package imports functions from the following CRAN/BioConductor packages: *BiocManager*, *Biobase*, *utils*, *methods*, *RSQLite*, *devtools*, *dplR*, *stringr*, *graphics*, *stats*, *affxparser*, and *data.table*. If it is desirable to pull datasets for GEO or perform the downstream analysis presented in this publication, the following additional packages are necessary: *siggenes*, *GEOquery*, and *R.utils*. All probe-level data and annotations utilized by the *GCSscore* package are parsed and sourced directly from the following chip specific BioConductor packages: platform design (.pd) and AnnotationDbi (.db). The resulting probe-level data file is packaged into a ‘probefile’ package, while the annotations are packaged into an additional ‘annot’ package. These packages are created on the fly and installed in the user’s library by utilizing customized versions of the *makeProbePackage* function and package templates sourced from the *AnnotationForge* package [16].

While the theory of the GCSscore method can be applied to any modern Affymetrix/Thermo-Fisher chip type, the R package was written for use with Clariom-style array, which include: ClariomS, ClariomD, and XTA assay chip types. This package fully supports all Clariom-style arrays and has support for two of the most widely used 3′ IVT arrays: Mouse Genome 430 2.0 and Human Genome UG133 2.0. For older types of Affymetrix arrays, the original *sscore* package must be used. The GCSscore algorithm allows the user to calculate GCS-score values for ClariomD/XTA arrays using two probe grouping methods: (1) utilizes TCids groupings for gene-level, (2) utilizes PSRids and JUCids for exon & alternate splicing-level. Since the ClariomS arrays only contain TCids, there is only a gene-level analysis method. Additionally, for supported 3′ IVT chips, the method refers to two background subtraction options: (1) utilizes the new GC-bkg method (2) utilizes the original PM-MM method.

The *GCSscore* package allows for direct probe-level comparisons of two Affymetrix microarrays at a time. The user can either input two. CEL files directly into the function, or read in a formatted batch file that is setup to run pair-wise comparisons of multiple. CEL files in a single function call (Additional File 1: Table S1). For more information regarding the implementation, please refer to the workflow diagram (Additional File 1: Figure S1). In brief:. CEL files are scaled to have equivalent trimmed median intensities for the desired probe grouping method. The GCS-scores are calculated and normalized using the middle 98% of the raw scores. Finally, normalized results are combined with the annotation information parsed from the Bioconductor repository and are returned to the user’s environment using the *Biobase* data structure, *ExpressionSet*. The user can also choose to save the GCS-score results to disk, as a. CSV file.

### Statistical properties and analysis of GCS-scores

One principal advantage of the GCSscore based method is the simple Gaussian-like statistics of the resulting output (Additional File 1: Figure S2). If no extreme differential expression exists between two. CEL files then the *GCSscore* output will have a mean of 0 and a SD of 1 [2]. Since, each run is essentially z-scored and normalized prior to output, each GCS-score becomes a representation of the standard deviation from the mean of a Gaussian-like distribution. Thus, the absolute values of GCS-scores greater than 1.8–2.0 are likely to be statistically significant and this can be determined by using statistical testing of biological replicates with correction for multiple testing, as done with the SAM method below.

### Workflow for generating differential expression for downstream analysis

In our standard implementation, all treatment samples were run against all control samples in a pairwise fashion. For example, if there are 3 replicates for the treatment and 3 replicates for the control group, there will be 9 total pairwise comparisons (Additional File 1: Table S1). The GCS-scores were averaged for each treatment sample against all 3 control samples, producing 3 averaged GCS-score values, one for each of the treatment samples. This was done to reduce noise with small sample sizes and to prevent over inflation of sample numbers that would occur from taking all of the pairwise comparisons into account [17]. Alternatively, random pairings of treatment/control samples can be used for generation of GCS-scores [5]. Multiple-testing correction of the GCS-score differential expression analysis can then be applied. For the statistical analyses presented in this publication, the averages of each treatment replicate against all of the control samples were used as the input into a 1-class Significance Analysis of Microarrays (SAM) analysis for multiple testing correction in identifying genes with GCS-score values statistically different from 0, as demonstrated in prior publications with the original Sscore algorithm [5, 9, 17]. The SAM algorithm used here was provided by the *siggenes* package from Bioconductor. More complex experimental designs implement multiple group testing in SAM or other appropriate statistical methods, such as LIMMA [18]. The average of these treatment replicate averages, denoted as AvgSs, was used as an additional stringent filter to decrease contributions from genes with exceedingly small fold-changes, as reported previously [5, 9, 17]. To determine significantly regulated gene lists, the following criteria were thus used: genes from the SAM output within a determined FDR cutoff (e.g. 0.0125–0.1) and genes who also have |AvgSs| > 1.8. In the Clariom-based array cases explored in parts B and C of the Results section, downstream analysis of the gene lists was performed using ToppFun suite for Gene Ontology/Functional enrichment analysis and Ingenuity Pathway Analysis (IPA) to find significantly altered signaling pathways. For the ToppFun analysis, only the gene symbols from the generated gene lists were input directly into the suite. For the IPA analysis, the TCids, AvgSs, and rawp values from the SAM output were used as the input.

## Results

### Comparison with original algorithm

**Fig. 1:**
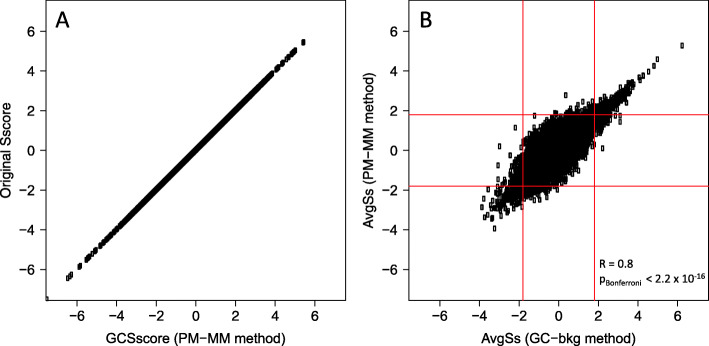
Comparison of GCSscore and Sscore methods. **a** Results for original Sscore algorithm (y-axis) and the GCSscore algorithm, using the PM-MM background correction method (x-axis). **b** Comparison of total averaged GCSscores (AvgSs) on the 3′ IVT array, Mouse Genome 430 2.0 assay, for the GC-based background correction (x-axis) and the legacy PM-MM method (y-axis). The red lines denote the thresholds for significant Sscores (|AvgSs| > 1.8)

Since the original Sscore algorithm has been validated and utilized in previous publications, we initially compared the GCSscore algorithm against the original Sscore method. In this example, previously published Mouse Genome 430 2.0 array data (GSE28515) from the prefrontal cortex of DBA2/J mice is used [9]. We utilized 3 biological replicates exposed to acute i.p. ethanol (1.8 g/kg × 4 h; treatment) and 3 biological replicates that received i.p. saline (control). The *GCSscore* package and algorithm was written to be much more efficient and contain several new functionalities compared with the original *sscore*. To confirm that the new algorithm performed as expected, we analyzed these 430 2.0 arrays first using the PM-MM method found in the *GCSscore* package with the original *sscore* package. For any given comparison between a treatment and a control, GCSscore (PM-MM method) and the original Sscore method produced identical GCS-score and S-score values (Fig. 1a), since the GCSscore algorithm simply utilizes individual MM probes instead of the GC-content based background (GC-bkg). The new GC-bkg method was also compared against the PM-MM method using the AvgSs metric described in the implementation section. There was considerable variation between the two methods as the AvgSs values approached zero, where there was no detectable difference between treatment and the control groups (Fig. 1b). However, convergence of the two methods was observed for GCS-score values beyond an empiric significance threshold of |AvgSs| > 1.8 (red lines in Fig. 1b). Previous studies have demonstrated that DBA2/J mice have many more genes that are upregulated than downregulated, especially in the medial prefrontal cortex (see figure 3 from [5]). This explains the dramatic skew towards positive significant AvgSs values, regardless of the background subtraction method. The results displayed in Fig. 1 demonstrate that the new background subtraction method returned very similar results to the original PM-MM method for significantly regulated genes, validating the use of the new GC-based background subtraction method.

### Comparison with RMA analysis of ClariomD/XTA assays

We then extended our characterization of *GCSscore* by comparing resulting analysis with a traditional RMA analysis for ClariomD/XTA arrays. In this use case, the GCS-score results were compared with RMA analysis results previously published in a study that utilizes ClariomD mouse arrays (aka MTA 1.0) to study effects of chronic diazepam (DZP) administration on gene expression in 3 mouse brain regions: cerebral cortex, hippocampus, and amygdala [19]. The. CEL files used in the GCSscore analysis were pulled directly from the corresponding GEO dataset: GSE76700. For the purposes of this analysis of the GCSscore algorithm, we limited comparisons to just cerebral cortex. There were 3 biological replicates in both the treatment (DZP) and control group. The original publication utilizes the standard workflow provided by Affymetrix’s Transcriptome Analysis Console (TAC). Using statistical criteria without correction for multiple testing (ANOVA uncorrected *p*-value ≤0.05 and |log2 fold change| > = 1.5), the authors identify 57 total transcripts regulated by chronic diazepam in cerebral cortex (see Suppl. Table 1 from [19]). Additionally, the reported PANTHER gene ontology enrichment results show over-representation only for general large categories such as “binding” or “receptor activity” (see Figure 1c from [19]). Only one gene, Lipocalin-2 (*Lcn2*), from the cortex results was replicated by quantitative PCR and used for further analysis, as it is the most strongly upregulated gene in all 3 brain regions investigated [19]. Current literature reports suggest that *Lcn2* is implicated in innate immune responses via the sequestration of iron.

**Fig. 2:**
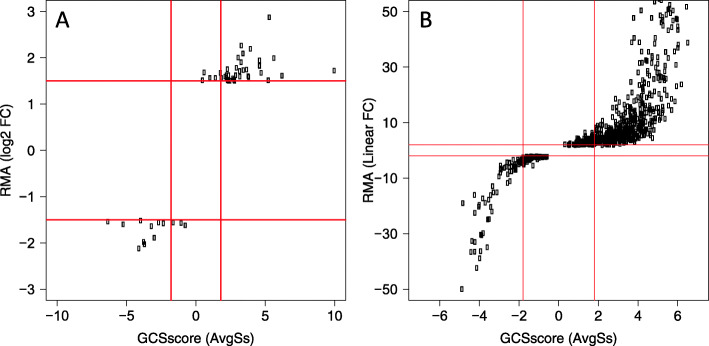
Fold change vs averaged GCS-score (AvgSs) values for significant TCids identified in the original publications. **a** Published significant TCids from GEO dataset: GSE76700. The vertical red lines denote the significant threshold for GCS-score values (|AvgSs| > 1.8). The horizontal lines denote the published threshold (FC > 1.5, *P* < 0.01). **b** Significant TCids from GEO dataset: GSE103380. The vertical red lines denote the significant threshold for GCS-score values (|AvgSs| > 1.8). The horizontal lines denote the published threshold (FC > 2, FDR < 0.05)

For the analysis of microarray data used in [19], GCS-scores for the 3 treated and 3 control. CEL files were generated in the pairwise fashion described in Implementation. This results in 3 treatment replicate averages produced for each of 3 treatment sample against all of the control samples. These 3 treatment replicate averages were used as the input for SAM statistical analysis. The GCSscore algorithm produced 432 transcripts with the following criteria: SAM-based FDR ≤ .015 and |AvgSs| > 1.8. Thus, the GCSscore method was able to produce many more significantly regulated transcripts with stringent multiple testing corrections, while the published RMA/LIMMA approach generated much smaller gene lists using uncorrected *p* values and modest fold changes produced from the standard TAC software. As expected, *Lcn2* (TC0200003303.mm.1) had very large positive GCS-scores for all comparisons, which resulted in an extremely high AvgSs = 17.21, replicating aspects of the results from the original publication. Strikingly, over-representation analysis of GCS-score results with ToppFun produced multiple categories directly related to diazepam/GABA biology (Table 1), as should be expected from the experimental paradigm. GO categories of note included: drug binding, glutamate decarboxylase activity, synaptic signaling, GABAergic synapse, and GABA synthesis. Pathway analysis of our GCSscore generated gene list via IPA produces multiple hits for immune-related signaling pathways, which supported the original findings from [19], in the context of the known functions of *Lcn2* (Additional File 1: Figure S3). Importantly, the top 20 pathways produced by IPA contained significant pathways for Glutamate degradation and GABA receptor signaling (Additional File 1: Figure S3). Furthermore, 50 of the 57 genes identified in [19] had a study-wide AvgSs > |1.8| and AvgSs values displayed a highly similar distribution (Fig. 2a) to the originally published log2 fold-change values (see Suppl. Table 1 from [19]).

**Table 1:** Functional enrichment for GCS-score results for dataset: GSE76700. Top GO categories returned from ToppFun suite

Category	ID	Name	*p*-value	FDR (B&H)
GO: Molecular Function	GO:0008144	drug binding	6.97E-05	2.11E-02
GO: Molecular Function	GO:0032559	adenyl ribonucleotide binding	8.10E-05	2.11E-02
GO: Molecular Function	GO:0030554	adenyl nucleotide binding	9.36E-05	2.11E-02
GO: Molecular Function	GO:0051020	GTPase binding	1.03E-04	2.11E-02
GO: Molecular Function	GO:0004351	glutamate decarboxylase activity	1.53E-04	2.13E-02
GO: Biological Process	GO:2001280	positive regulation of unsaturated fatty acid biosynthetic process	4.16E-06	1.91E-02
GO: Biological Process	GO:0045723	positive regulation of fatty acid biosynthetic process	1.62E-05	1.96E-02
GO: Biological Process	GO:0045923	positive regulation of fatty acid metabolic process	1.63E-05	1.96E-02
GO: Biological Process	GO:0099536	synaptic signaling	1.79E-05	1.96E-02
GO: Biological Process	GO:0098916	anterograde trans-synaptic signaling	3.25E-05	1.96E-02
GO: Cellular Component	GO:0030136	clathrin-coated vesicle	6.26E-07	3.52E-04
GO: Cellular Component	GO:0045202	synapse	1.20E-05	2.68E-03
GO: Cellular Component	GO:0030135	coated vesicle	1.43E-05	2.68E-03
GO: Cellular Component	GO:0005938	cell cortex	2.67E-05	3.76E-03
GO: Cellular Component	GO:0030054	cell junction	5.85E-05	5.70E-03
Pathway	1,268,766	Transmission across Chemical Synapses	2.54E-06	3.70E-03
Pathway	P00018	EGF receptor signaling pathway	2.70E-05	1.62E-02
Pathway	377,263	GABAergic synapse	3.34E-05	1.62E-02
Pathway	M8353	Human Cytomegalovirus and Map Kinase Pathways	7.95E-05	2.90E-02
Pathway	1,268,763	Neuronal System	1.37E-04	3.30E-02
Pathway	83,105	Pathways in cancer	1.52E-04	3.30E-02
Pathway	137,938	IL2 signaling events mediated by PI3K	1.61E-04	3.30E-02
Pathway	1,268,775	GABA synthesis	2.02E-04	3.30E-02
Drug	ctd: D003024	Clozapine	4.37E-11	1.06E-06
Drug	ctd: D020849	Raloxifene Hydrochloride	2.07E-10	2.01E-06
Drug	ctd: D004390	Chlorpyrifos	2.49E-10	2.01E-06
Drug	ctd: C548651	2-(1’H-indolo-3′-carbonyl)thiazole-4-carboxylic acid methyl ester	3.55E-09	2.15E-05
Drug	CID000005637	U0126	9.57E-09	4.65E-05
Disease	C0027051	Myocardial Infarction	1.98E-06	6.85E-03
Disease	C0006012	Borderline Personality Disorder	1.35E-05	2.29E-02
Disease	C0917798	Cerebral Ischemia	2.32E-05	2.29E-02
Disease	C0006287	Bronchopulmonary Dysplasia	2.65E-05	2.29E-02
Disease	C0001430	Adenoma	5.34E-05	3.69E-02
Disease	C0001973	Alcoholic Intoxication, Chronic	7.18E-05	4.14E-02

The original publication also investigates *Lcn2* at the exon-level using the differential splicing functionality of the TAC software. The MTA 1.0 array has 15 PSRids (8 targeting exons) and 5 JUCids targeting splice junctions for the *Lcn2* transcript (see Table 2). The authors of the original publication deduce that the main transcript, Lcn2–201, is up-regulated rather than the only other protein-coding transcript variant, Lcn2–206 (see Figure 4.A from [19]).

**Table 2:** GCS-score results for all 15 exon-level probesetids assigned to *Lcn2*. Probesetids in bold are either within an exon (PSR) or connect 2 exons (JUC)

Probesetid	Exon ID	GCSscore
**PSR0200028082.mm.1**	**Exon 6**	**4.201**
**PSR0200028083.mm.1**	**Exon 6**	**8.35**
PSR0200028084.mm.1	intron	−0.15
PSR0200028085.mm.1	intron	0.27
**PSR0200028086.mm.1**	**Exon 5**	**9.1**
PSR0200028087.mm.1	intron	−0.44
**PSR0200028088.mm.1**	**Exon 4**	**9.92**
PSR0200028089.mm.1	intron	0.83
**PSR0200028090.mm.1**	**Exon 3**	**8.4**
**PSR0200028091.mm.1**	**Exon 3**	**9.68**
PSR0200028092.mm.1	intron	−1.585
PSR0200028093.mm.1	intron	−0.071
**PSR0200028094.mm.1**	**Exon 2**	**9.26**
**PSR0200028095.mm.1**	**Exon 1**	**5.17**
PSR0200028098.mm.1	upstream intron	−0.42
**JUC0200014167.mm.1**	**Exon 5 -- Exon 6**	**5.1**
**JUC0200014163.mm.1**	**Exon 4 -- Exon 5**	**5.97**
**JUC0200014164.mm.1**	**Exon 3 -- Exon 4**	**6.19**
**JUC0200014165.mm.1**	**Exon 2 -- Exon 3**	**2.65**
**JUC0200014166.mm.1**	**Exon 1 -- Exon 2**	**4.42**

**Fig. 3:**
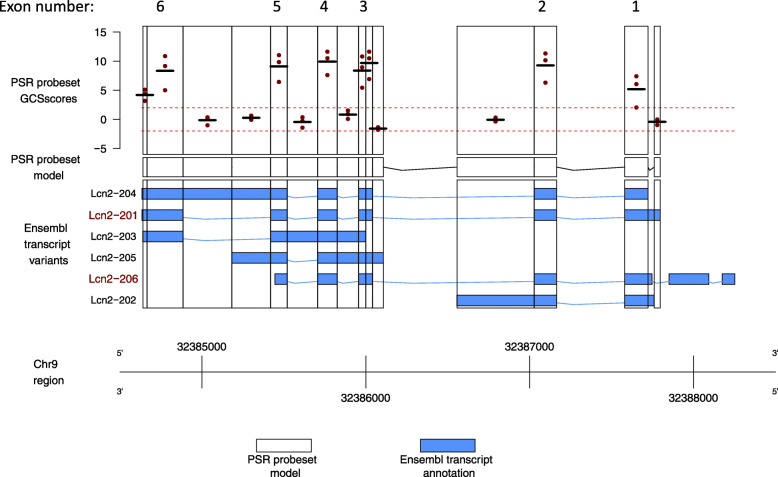
GCS-scores for all PSRids assigned to *Lcn2.* The coverage segments of each PSRid are shown in the ‘PSR probeset model’ and in the highlighted segments of ‘PSR probeset GCSscores’ and ‘Ensembl transcript variants’. Ensembl transcripts that are ‘protein-coding’ are in red (Lcn2–201 and Lcn2–206)

**Fig. 4:**
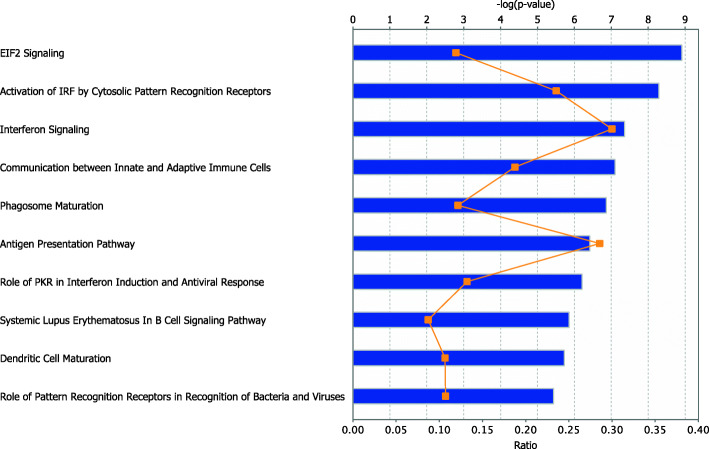
Top 10 enriched pathways via Ingenuity Pathway Analysis (IPA) for GCSscore analysis of GEO dataset: GSE103380

Using the exon-level GCSscore method to analyze *Lcn2*, we found similar results to the original publication. The GCSscore method found that all 8 PSRids targeting exons were significantly upregulated, while none of the PSRids that targeted introns were altered (see Table 2 and Fig. 3). Furthermore, all 5 of the JUCids were significantly upregulated in the GCS-score results. The genomic location of PSRids targeting introns suggest that the transcripts with retained introns (Lcn-202 to Lcn2–205) are unlikely to be regulated by the treatment (see Figure 4.A from [19]). Additionally, both PSRids in the final exon of Lcn-201 (exon 6), are significantly upregulated via the GCSscore method. Since exon 6 is not found in Lcn2–206, we could not fully eliminate the possibility of Lcn2–206 upregulation, but we confidently concluded that the main transcript, Lcn2–201, was upregulated and that *Lcn2* variants with retained introns were unlikely to be altered by diazepam treatment. This demonstrates the utility of GCSscore exon-level method for deducing which transcript variants are altered for the significantly regulated genes identified by the GCSscore gene-level method. These results implied that the *GCSscore* package a valuable tool for both detecting significantly regulated genes and differential splicing analysis of exons for ClariomD/XTA type arrays.

### Comparison with RMA analysis of ClariomS assays

A final comparison illustrates use of the GCSscore vs. RMA/LIMMA methods for analysis of published results with the ClariomS platform. As described in [20], the original study utilizes ClariomS mouse arrays to investigate differential gene expression of mouse microglia cells 4 days after infection with the coronavirus, murine hepatitis virus (MHV). In this study, the authors generate a gene list using an FDR cutoff of 0.05 and a linear fold change with an absolute value greater than 2. The authors utilize IPA to produce enriched pathways for their significant genes (see figure 1.C from [20]). The authors find that interferon (IFN) signaling is the most upregulated pathway following infection, followed by 3 additional pathways linked directly to the immune system [20]. They also report the expression metrics of 29 select genes from their gene list that were highly upregulated (see figure 1.D from [20]). All microarray data related to the microglial analysis in [20] is available in GEO dataset: GSE103380.

For GCSscore analysis, all 4 naïve (control) samples and all 4 infected samples in the GEO dataset were investigated, leading to 16 total pairwise comparisons of CEL files. As described above, the resulting 4 treatment replicate averages were interrogated by a 1-class SAM analysis to detect GCS-scores ≠ 0. This resulted in 486 genes that passed the determined selection criteria (FDR < 0.0125 and |AvgSs| > 1.8). The resulting gene list was input into both IPA and ToppFun for functional over-representation analysis as described in the Implementation section. The IPA analysis produced multiple pathways related to immune function, including the top pathways found in the original publication (Fig. 4). Of note, all top 10 pathways from the GCSscore analysis are related directly to immune response and function, which is an even stronger implication of the biological functions observed in the original published analysis (see Figure 1.C from [20]). Furthermore, 2 of the enriched pathways unique to the GCSscore results, “eIF2 Signaling” and “role of PKR in Interferon Induction and Antiviral Response”, are likely upstream of the interferon signaling pathways identified in both methods. Recent literature has demonstrated that eIF2-alpha is integral for maximum production of inflammatory cytokines and type I interferons in response to microbial infection [21]. This suggested that the GCSscore method was also able to identify potential important additional biological functions related to this experimental design. Furthermore, the ToppFun analysis was consistent with the IPA data, showing major enrichment for categories related to immune response and modulating interferon production during a viral infection (Table 3). Additionally, AvgSs values displayed a high degree of correlation (see Fig. 2b) with the linear fold change values for significantly regulated genes identified by the authors of the original manuscript (S. Perlman, personal communication; data not shown). Finally, 24 of the 29 (83%) of the selected upregulated genes highlighted in the original publication (Figure 1.D from [15]) were also contained in the GCSscore derived gene list, demonstrating good overlap between the methods when the comparing the most differentially regulated transcripts that are identified by either method.

**Table 3:** Functional enrichment for GCS-score results for dataset: GSE103380. Top GO categories returned from ToppFun Suite

Category	ID	Name	*p*-value	FDR (B&H)
GO: Molecular Function	GO:0003723	RNA binding	1.11E-10	1.18E-07
GO: Molecular Function	GO:0017076	purine nucleotide binding	5.01E-08	2.16E-05
GO: Molecular Function	GO:0003735	structural constituent of ribosome	6.07E-08	2.16E-05
GO: Molecular Function	GO:0032555	purine ribonucleotide binding	1.48E-07	3.93E-05
GO: Molecular Function	GO:0032553	ribonucleotide binding	2.09E-07	4.45E-05
GO: Biological Process	GO:0044403	symbiotic process	1.16E-35	6.36E-32
GO: Biological Process	GO:0016032	viral process	2.25E-35	6.36E-32
GO: Biological Process	GO:0044419	interspecies interaction between organisms	1.03E-34	1.93E-31
GO: Biological Process	GO:0051707	response to other organism	2.90E-32	3.06E-29
GO: Biological Process	GO:0009607	response to biotic stimulus	3.08E-32	3.06E-29
GO: Biological Process	GO:0043207	response to external biotic stimulus	3.25E-32	3.06E-29
GO: Biological Process	GO:0098542	defense response to other organism	9.85E-32	7.95E-29
GO: Biological Process	GO:0045087	innate immune response	3.58E-31	2.53E-28
GO: Biological Process	GO:0006952	defense response	4.28E-28	2.69E-25
GO: Biological Process	GO:0002252	immune effector process	3.18E-26	1.80E-23
GO: Cellular Component	GO:0022626	cytosolic ribosome	1.48E-15	1.01E-12
GO: Cellular Component	GO:0005840	ribosome	2.06E-10	5.84E-08
GO: Cellular Component	GO:0044391	ribosomal subunit	2.57E-10	5.84E-08
GO: Cellular Component	GO:0005764	lysosome	4.85E-10	6.90E-08
GO: Cellular Component	GO:0000323	lytic vacuole	5.05E-10	6.90E-08
Mouse Phenotype	MP:0005025	abnormal response to infection	5.88E-18	1.32E-14
Mouse Phenotype	MP:0001793	altered susceptibility to infection	7.44E-18	1.32E-14
Mouse Phenotype	MP:0002406	increased susceptibility to infection	3.38E-16	4.01E-13
Mouse Phenotype	MP:0020185	altered susceptibility to viral infection	1.27E-14	1.13E-11
Mouse Phenotype	MP:0002418	increased susceptibility to viral infection	2.17E-14	1.55E-11
Pathway	1,269,311	Interferon Signaling	1.95E-19	3.36E-16
Pathway	1,269,310	Cytokine Signaling in Immune system	4.81E-15	3.38E-12
Pathway	1,269,312	Interferon alpha/beta signaling	5.86E-15	3.38E-12
Pathway	1,269,108	Influenza Infection	3.72E-14	1.61E-11
Pathway	1,268,686	GTP hydrolysis and joining of the 60S ribosomal subunit	3.13E-13	7.91E-11
Disease	C0023893	Liver Cirrhosis, Experimental	1.08E-13	4.66E-10
Disease	C0042769	Virus Diseases	1.34E-12	2.89E-09
Disease	C0021400	Influenza	7.78E-12	9.47E-09
Disease	C0024141	Lupus Erythematosus, Systemic	8.76E-12	9.47E-09
Disease	C0019196	Hepatitis C	4.01E-11	2.90E-08

## Discussion

Here we have described a new software methodology for analysis of the latest generation of Affymetrix/Thermo-Fisher microarrays, based upon a re-derivation of our original Sscore algorithm that allows analysis of current oligonucleotide microarray platforms. Since microarray technology has inherent advantages for certain genomic studies compared to RNAseq, on the basis of costs and time required for analysis, such methodology development is of considerable significance. Furthermore, microarray studies continue to be utilized for thousands of publications in the published literature each year, thus documenting a sizeable ongoing scientific contribution. Our software development is an outgrowth of the original Sscore method for probe-level analysis of Affymetrix arrays which has been previously validated as a sensitive method for differential gene expression analysis that was particularly valuable for studies employing low numbers of replicates [2, 3]. Here we have presented three use cases to demonstrate that our new R package software and analysis algorithm, *GCSscore*, can provide analysis results equal to the prior Sscore method for older generation (3′ IVT) microarrays, while delivering substantial benefits compared to existing methods for analysis of the newest ClariomS/ClariomD/XTA arrays.

We demonstrated that for 3′ IVT arrays, GCSscore produced very similar differential gene expression analysis results compared to the original Sscore method, which had been validated against multiple other algorithms, including RMA [3]. We also found that the GCSscore method produced similar results to the existing RMA/LIMMA method for the ClariomS arrays. The results presented here suggest that GCSscore provides greater sensitivity for detection of DEG gene lists for the ClariomS assay, as evidenced by results of the IPA and ToppFun enrichments. In particular, the GCSscore method may provide more relevant pathways, including two potential upstream regulators of the interferon signaling identified in [20]. Additionally, *GCSscore* produced a larger gene set than the methods used in [15] when using similar statistical thresholds (S. Perlman, personal communication; data not shown). Finally, we showed that GCSscore was far superior to the traditional RMA/LIMMA approach for analysis of ClariomD/XTA based studies. The GCSscore algorithm was able to identify many significantly regulated transcripts that survived multiple test correction, while the RMA/LIMMA method returned very few transcripts, even with uncorrected *p*-values. Importantly, the GCSscore method led to increased biological insight that was consistent with the studied treatment, as evidenced by identification of multiple gene sets over-represented with functional groups and pathways related to GABA biology, as would be expected when profiling the cortex of chronically diazepam-treated animals. In addition, the GCSscore exon-level analysis was capable of providing critical details regarding the regulation of individual transcript variants in genes that show significant regulation at the gene-level.

Although not the object of this report, it is peculiar that the RMA/LIMMA methods appeared to work satisfactorily with ClariomS platforms, but failed to function sensitively for ClariomD/XTA assays. The ClariomS array is derived from the accompanying ClariomD/XTA array for each species (mouse, rat, human). In fact, multiple publications that use ClariomD/XTA arrays only report uncorrected *p*-values due to this limitation [19, 22]. The ClariomS arrays are composed of probes taken directly from the corresponding ClariomD/XTA array but utilizes only the 10 best performing probes for TCids that code for well annotated genes producing at least one protein-coding variant. Thus, ClariomD/XTA arrays target many more noncoding transcripts than protein-coding transcripts. Non-coding TCids on ClariomD/XTA arrays are the predominant probe type and tend to be expressed at very low or high levels compared to protein-coding gene probes (data not shown). We suggest that the LIMMA analysis of log2 RMA intensities from ClariomD/XTA arrays may be affected by the overwhelming number of noncoding transcripts found in these low and high intensity distributions. This disparate signal distribution might alter the normalization of RMA intensity results and thus increase signal/noise variance and thereby reduce statistically significant results from FDR analysis of these arrays. GCSscore methods are immune to this effect since it only considers the relative changes for each individual TCid/PSRid when making comparisons. These individual TCid/PSRid comparisons are independent of each other, so the coding transcripts are unaffected by the noncoding transcripts. Furthermore, we demonstrated that *GCSscore* is able to produce significant results for the ClariomD/XTA arrays with as few as 3 control and 3 treatment arrays, which reduces the time and costs of genomic experiments that utilize this technology. These properties make the GCSscore method a powerful analysis tool for the most advanced array technology available on ClariomD/XTA arrays, as well as the ClariomS arrays. The new methodology described here also further supports the inherent strengths of the probe-level analysis provided by the GCSscore algorithm. Future utilization of GCSscore and direct comparison with other microarray analysis approaches would further add to an understanding of the merits for such probe-level strategies and may impact methodology development for other transcriptomic approaches such as RNAseq.

## Conclusions

The *GCSscore* package represents a powerful new application for analysis of the newest generation of oligonucleotide microarrays such as the ClariomS and ClariomD/XTA arrays produced by Affymetrix/Fisher. Based upon a well-validated legacy platform, *sscore*, this new software allows production of increased scientific insight from the latest microarray genomic analysis platforms.

## Availability and requirements

All datasets used in this publication are freely available in the GEO database (GSE28515, GSE76700, GSE103380).

**Package name:** GCSscore

**Package home page:** https://github.com/harrisgm/GCSscore or http://www.bioconductor.org/packages/release/bioc/html/GCSscore.html. The Bioconductor home page includes the source files, the compiled executables and a software primer with demo examples.

**Operating System:** Any OS that supports the R programming Language, including: Windows, macOS, and Unix-based systems.

**Programming Language:** R.

**Other Requirements:** ability to compile R packages. For Windows, install Rtools. For macOS, install xcode command line options. See README on GitHub home page for more details.

**Recommended Hardware:** The memory requirements to run GCSscore is minimal as. CEL files are loaded individually and the annotation packages are relatively small. However, there is greater memory usage when building the probefile and the annotation packages for ClariomD/XTA chips. It is recommended that the user has at least 8GB of RAM, but 16GB of RAM is the recommended amount. The GCSscore computations were performed on a computer with 32GB of RAM and a 2.9GHz Intel i9 processor with 6 cores.

**License:** GNU GPL version 3.

**Restrictions for use by non-academics:** None.

## Supplementary Information

**Additional file 1: Table S1:** Example of batch.csv file structure used for batch input into *GCSscore* package. **Figure S1:** Workflow diagram for GCS-score algorithm. **Figure S2:** Example of typical GCS-score histogram. This histogram was derived from an an exon-level (PSRid/JUCid) analysis of two MTA 1.0 CEL files. **Figure S3:** Functional pathway enrichment for GCS-score results for GEO dataset: GSE76700. Top 20 pathways returned from IPA.

## Abbreviations

DEG: Differentially Expressed Genes
RMA: Robust MultiChip Average
SAM: Significance Analysis of Microarrays
LIMMA: Linear Models for Microarray Data
IPA: Ingenuity Pathway Analysis
GC-bkg: GC-content based background subtraction
FC: Fold Change
NSB: Non-specific binding

## References

[1] Irizarry RA, Hobbs B, Collin F, Beazer-Barclay YD, Antonellis KJ, Scherf U (2003). Exploration, normalization, and summaries of high density oligonucleotide array probe level data. Biostatistics..

[2] Zhang L, Wang L, Ravindranathan A, Miles MF (2002). A new algorithm for analysis of oligonucleotide arrays: application to expression profiling in mouse brain regions. J Mol Biol.

[3] Kennedy RE, Archer KJ, Miles MF (2006). Empirical validation of the S-score algorithm in the analysis of gene expression data. BMC Bioinformatics.

[4] Kennedy RE, Kerns RT, Kong X, Archer KJ, Miles MF (2006). SScore: an R package for detecting differential gene expression without gene expression summaries. Bioinformatics..

[5] Kerns RT, Ravindranathan A, Hassan S, Cage MP, York T, Sikela JM (2005). Ethanol-responsive brain region expression networks: implications for behavioral responses to acute ethanol in DBA/2J versus C57BL/6J mice. J Neurosci.

[6] Grice DE, Reenilä I, Männistö PT, Brooks AI, Smith GG, Golden GT (2007). Transcriptional profiling of C57 and DBA strains of mice in the absence and presence of morphine. BMC Genomics.

[7] Singh SK, Bhardwaj R, Wilczynska KM, Dumur CI, Kordula T (2011). A complex of nuclear factor I-X3 and STAT3 regulates astrocyte and glioma migration through the secreted glycoprotein YKL-40. J Biol Chem.

[8] Wolstenholme JT, Warner JA, Capparuccini MI, Archer KJ, Shelton KL, Miles MF (2011). Genomic analysis of individual differences in ethanol drinking: evidence for non-genetic factors in C57BL/6 mice. PLoS One.

[9] Wolen AR, Phillips CA, Langston MA, Putman AH, Vorster PJ, Bruce NA (2012). Genetic dissection of acute ethanol responsive gene networks in prefrontal cortex: functional and mechanistic implications. PLoS One.

[10] Paxson JA, Gruntman AM, Davis AM, Parkin CM, Ingenito EP, Hoffman AM (2013). Age dependence of lung mesenchymal stromal cell dynamics following pneumonectomy. Stem Cells Dev.

[11] Van Der Vaart AD, Wolstenholme JT, Smith ML, Harris GM, Lopez MF, Wolen AR (2017). The allostatic impact of chronic ethanol on gene expression : a genetic analysis of chronic intermittent ethanol treatment in the BXD cohort. Alcohol..

[12] Bogenpohl JW, Smith ML, Farris SP, Dumur CI, Lopez MF, Becker HC (2019). Cross-species co-analysis of prefrontal cortex chronic ethanol transcriptome responses in mice and monkeys. Front Mol Neurosci.

[13] Schuster EF, Blanc E, Partridge L, Thornton JM (2007). Estimation and correction of non-specific binding in a large-scale spike-in experiment. Genome Biol.

[14] Affymetrix Exon Array Background Correction White Paper. Revised 9/27/2005. http://tools.thermofisher.com/content/sfs/brochures/exon_background_correction_whitepaper.pdf.

[15] Xu W, Seok J, Mindrinos MN, Schweitzer AC, Jiang H, Wilhelmy J (2011). Human transcriptome array for high-throughput clinical studies. Proc Natl Acad Sci U S A.

[16] Carlson M, Obenchain V (2015). AnnotationForge: Tools for building SQLite-based annotation data packages. Bioconductor.

[17] Farris SP, Miles MF (2013). Fyn-dependent gene networks in acute ethanol sensitivity. PLoS One.

[18] Ritchie ME, Phipson B, Wu D, Hu Y, Law CW, Shi W (2015). Limma powers differential expression analyses for RNA-sequencing and microarray studies. Nucleic Acids Res.

[19] Furukawa T, Shimoyama S, Miki Y, Nikaido Y, Koga K, Nakamura K (2017). Chronic diazepam administration increases the expression of Lcn2 in the CNS. Pharmacol Res Perspect.

[20] Wheeler DL, Sariol A, Meyerholz DK, Perlman S (2018). Microglia are required for protection against lethal coronavirus encephalitis in mice. J Clin Invest.

[21] Pierre P (2019). Integrating stress responses and immunity. Science (80- ).

[22] Wolstenholme JT, Mahmood T, Harris GM, Abbas S, Miles MF (2017). Intermittent ethanol during adolescence leads to lasting behavioral changes in adulthood and alters gene expression and histone methylation in the PFC. Front Mol Neurosci.

