# Supplementary material for: GCSscore: an R package for differential gene expression analysis in Affymetrix/Thermo-Fisher whole transcriptome microarrays
Source: BMC Genomics. 2021 Feb 1;22:96. doi: 10.1186/s12864-021-07370-2 (PMC7848880; doi:10.1186/s12864-021-07370-2)
Supplement: Supplementary file 1 — Additional file 1: Table S1: Example of batch.csv file structure used for batch input into GCSscore package. Figure S1: Workflow diagram for GCS-score algorithm. Figure S2: Example of typical GCS-score histogram. This histogram was derived from an an exon-level (PSRid/JUCid) analysis of two MTA 1.0 CEL files. Figure S3: Functional pathway enrichment for GCS-score results for GEO dataset: GSE76700. Top 20 pathways returned from IPA. [file 12864_2021_7370_MOESM1_ESM.docx]

Supplementary Information:

**Table S1:** Example of batch.csv file structure used for batch input into *GCSscore* package.

**Figure S1:** Workflow diagram for GCS-score algorithm.

**Figure S2:** Example of typical GCS-score histogram. This histogram was derived from an an exon-level (PSRid/JUCid) analysis of two MTA 1.0 CEL files.

**Figure S3:** Functional pathway enrichment for GCS-score results for GEO dataset: GSE76700. Top 20 pathways returned from IPA.
